# The Acquisition of /ɪ/–/iː/ Is Challenging: Perceptual and Production Evidence from Cypriot Greek Speakers of English

**DOI:** 10.3390/bs12120469

**Published:** 2022-11-22

**Authors:** Georgios P. Georgiou

**Affiliations:** Department of Languages and Literature, University of Nicosia, Nicosia 2417, Cyprus; georgiou.georg@unic.ac.cy

**Keywords:** speech perception, speech production, second language, vowels, Cypriot Greek, English, Universal Perceptual Model

## Abstract

This study aims to investigate the perception and production of the English /ɪ/–/iː/ vowel contrast by Cypriot Greek speakers of English as a second language (L2). The participants completed a classification test in which they classified the L2 vowels in terms of their first language (L1) categories, a discrimination test in which they distinguished the members of the vowel contrast, and a production test in which they produced the target vowels. The results showed that they classified both L2 /ɪ/–/iː/ mostly in terms of L1 /i/, which denotes the formation of a completely overlapping contrast according to the theoretical framework of the Universal Perceptual Model (UPM), and that they could hardly distinguish the vowel pair. In addition, their productions deviated in most acoustic parameters from the corresponding productions of English controls. The findings suggest that /ɪ/–/iː/ may carry a universal marker of difficulty for speakers with L1s that do not possess this contrast. This distinction is difficult even for experienced L2 speakers probably because they had never been exposed to naturalistic L2 stimuli and they do not use the L2 that much in their daily life. Finally, the study verifies UPM’s predictions about the discriminability of the contrast and extends the model’s implications to speech production; when an L2 vowel contrast is perceived as completely overlapping, speakers activate a (near-) unified interlinguistic exemplar in their vowel space, which represents both L2 vowels.

## 1. Introduction

The inadequacy of adult speakers in accurately perceiving the sounds of a second language (L2) has been described in many recent studies [1,2,3,4,5,6,7,8,9]. It has been proposed that this difficulty results from continuous exposure to a specific language (i.e., first language; L1) that changes the way non-native speech sounds are perceived [10]. Problematic perception is believed to lead to problematic production [11,12,13], which appears in the form of accented speech [14,15,16,17,18]. This is particularly evident for sounds or sound contrasts that are absent from the speakers’ L1 [19,20]. There is a need to examine the acquisition of L2 contrasts by listeners with various—including under-researched—varieties to better define the speech acquisition processes and mechanisms. This study aims to investigate the perception and production of the English /ɪ/–/iː/ vowel contrast by Cypriot Greek (henceforth, CG) speakers of L2 English.

The previous findings have suggested that perceptual difficulties are attributed to the size and complexity of the L1–L2 vowel systems [21,22,23]. Iverson and Evans [22] found that German and Norwegian speakers of English can recognize English vowels easier than Spanish and French speakers of English. This is because the L1 vowel system of the former speakers is larger and more complex than that of English, while this is not the case for the L1 vowel system of the latter speakers. However, more recent studies have questioned the sole role of inventory size and complexity, indicating that *acoustic–phonetic* differences between the sounds of the L1–L2 vowel systems are critical for the prediction of perceptual difficulties [24,25]. For example, Alispahic and colleagues [24] found that Dutch vowel contrasts were not discriminated better by Australian English than Peruvian Spanish listeners, although the Australian English vowel system is as large as the Dutch one, while the Peruvian Spanish vowel system is much smaller. Thus, the acoustic–phonetic characteristics of L1 and L2 sounds may also define the perception of L2 sounds.

Speech acquisition studies are often developed on the basis of a theoretical model. Among the most important and widely used models are the *Speech Learning Model* (SLM) [26] and its revised version, *SLMr* [27], the *Perceptual Assimilation Model* (PAM) [28] and its extension *PAM-L2* [29], and the *Second Language Linguistic Perception* model (L2LP) [30]. The *Universal Perceptual Model of Second Language* (UPM) [31] was recently developed to account for the perceptual difficulties of L2 learners. The model supports that there is a universal capacity for humans to learn non-native sounds across the lifespan. Speakers extract information from the speech signal and activate phonetic categories (i.e., mental representations of speech sounds). Non-native speech sounds are usually *disoriented* but they can be oriented toward native productions under specific circumstances (e.g., if speakers use the L2 consistently, if they receive phonetic training, etc.). The perceptual predictions of UPM are developed on the basis of crosslinguistic *perceptual similarity*. Two L2 sounds might be *completely overlapping* (that is, sharing the same above chance L1 response or the same set of L1 responses), *partially overlapping* (that is, sharing at least one above chance response), and *nonoverlapping* (that is, not sharing any above chance responses). Completely overlapping contrasts (similar to the Single Category assimilation of PAM) are expected to have the poorest discrimination, followed by partially overlapping (similar to the Category Goodness assimilation of PAM) and nonoverlapping contrasts (similar to the Two Category assimilation of PAM), which are predicted to have the best discrimination accuracy. The predictions of UPM have been investigated in two recent studies. Georgiou [31] found that the model could predict with success the discrimination accuracy of Italian contrasts by CG learners of Italian. In addition, Georgiou and colleagues [32] observed that UPM successfully predicted the discriminability of several L2 English contrasts by Greek monolingual and bidialectal speakers.

The acquisition of English /ɪ/–/iː/ has been investigated in several studies, considering that it comprises a challenging contrast for speakers whose language does not have such a distinction. Concerning the perceptual domain, Georgiou [33], who examined the vowel perceptual patterns of CG child learners of English, found that English vowels /ɪ/ and /iː/ were assimilated to the same L1 category (i.e., CG /i/) by both the lowly and the highly experienced learners and that the discrimination accuracy of this contrast was just fair. In another study, Morrison [34] concluded that Spanish listeners of Canadian English cannot distinguish English /ɪ/–/iː/ at the initial stage since both English vowels have a close spectral range with Spanish /i/ and, therefore, listeners assimilate them to that L1 category. Yang and colleagues [35] assessed the ability of Chinese college students to perceive the synthetic American English /i/–/ɪ/ vowel contrast. The results showed that the students employed different perceptual patterns (i.e., reliance only on temporal cues) compared to native speakers. This is because both English vowels were perceived as being equivalent to Mandarin /i/, thereby creating perceptual confusion. With respect to the production domain, Cebrian [36] examined the production of English vowels /i/ and /ɪ/ by Catalan speakers among other. The author claimed that the members of the /i/–/ɪ/ contrast were produced with an acoustic overlap, demonstrating the difficulty of speakers in articulating these vowels. Fullana-Rivera and MacKay [37] explored the production of English /ɪ/–/i/ by Spanish EFL learners, reporting that the production of /ɪ/ differed from the corresponding production of English native speakers, while the production of /i/ did not. The problematic production of the former English vowel may have resulted from the fact that this vowel is not part of the Spanish system. In contrast to the previous studies, speakers of other languages such as German, do not have significant difficulties with the acquisition of English /ɪ/–/iː/. For example, Strange and colleagues [38] found that the American English vowels /ɪ/ and /iː/ were categorized into the L1 North German /ɪ/ and /iː/, respectively, indicating no difficulty for the German speakers. This is because the aforementioned English vowels are present in the German vowel system, creating contrast with each other. In addition, Llompart and Reinisch [39] reported that English /ɪ/–/iː/ was an easy contrast for German speakers as it noted robust perception and better differentiation during production compared to /ɛ/–/æ/.

Speech perception is linked to speech production and, therefore, any perceptual deficits are expected to also appear in production. Several psycholinguistic theories attempt to define this link. The motor theory [40,41] suggests that speech perception occurs by detecting intended vocal tract gestures rather than spoken speech. These gestures act as motor commands, providing certain instructions to the articulators. Another important theory is direct realism [42], which proposes that there is a direct perception of speakers’ articulatory gestures (objects) and that speakers attempt to use these gesturally defined objects in speech production—speech perception and production share a common communicative goal [43]. In an attempt to define the perception–production link, Georgiou [44] investigated the relationship between perceived crosslinguistic similarity and L2 production. The participants were Egyptian Arabic learners of L2 Greek who participated in perceptual and production tests. The results demonstrated that some assimilation patterns could be reflected in production, e.g., the assimilation of CG /i/ and /e/ to a single L1 category led to the production of these vowels with partial overlap. In addition, most of the learners’ production patterns could be predicted by the classification test. Further evidence about this link is provided by the results of several training studies that portray transfer effects from perception to production [43,45,46]. Georgiou [45] trained CG adult and child learners of English in the discrimination of English vowel contrasts. The author found that perceptually oriented phonetic training helped children not only to improve their vowel perception patterns but also to improve to some extent their production patterns—this provides evidence for a common mental space for speech perception and production. Nevertheless, some studies indicate no such transfer (e.g., [47]).

The present study aims to answer the following research questions: (i) how do CG speakers of L2 English classify English vowels /ɪ/ and /iː/ in terms of their L1 phonetic categories?; (ii) how do they distinguish these vowels?; (iii) is UPM capable of producing accurate predictions regarding the discrimination of this vowel contrast?; (iv) and what are the acoustic characteristics of English /ɪ/ and /iː/ (*F1*, *F2*, and duration) and how do these compare with those of English native speakers? To the best of our knowledge, this is the first study focusing on the acquisition of this English contrast by CG speakers in both the perceptual and the production domains. We will employ for the first time the framework of UPM to examine how the perceptual predictions can extend to production. Considering the lack of this contrast in CG and its dense vowel system (it consists of vowels /i e a o u/), we hypothesize that English /ɪ/ and /iː/ will both be perceived as instances of CG /i/. We expect to see a high overlap in the classification of these vowels since they are acoustically close to CG /i/ and, therefore, the discrimination is expected to be poor. In addition, we expect that they will be produced in a non-native-like manner since there are acoustic–phonetic differences between English /ɪ/ and /iː/ and their acoustically closer exemplar, CG /i/.

## 2. Formulation of Predictions

To form the predictions about the classification of English /ɪ/ and /iː/ in terms of CG vowel categories and the discrimination accuracy of this contrast, we developed an acoustic study, which compared the acoustic characteristics of English and CG vowels. The motivation for such a study lies in the previous success of crosslinguistic acoustic analyses in predicting L2 vowel perceptual patterns.

**Figure 1:**
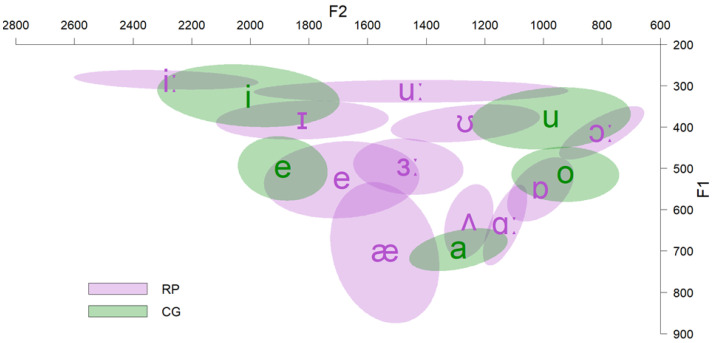
*F1 × F2* of CG and RP vowels.

The data were gathered from previous acoustic analyses. Ten adult native speakers of English (Received Pronunciation; RP) were asked to produce their native vowels in an /hVd/ (V = vowel) word context (11 vowels × 10 speakers = 110 productions). Words were part of the carrier phrase “they say < target word > now”. Another 11 native speakers of CG produced their native vowels in an /pVs/ word context, embedded within the carrier phrase ‘Léne < target word > tóra’ (5 vowels × 11 speakers × 2 repetitions = 110 productions). All speakers were instructed to produce the stimuli as if speaking to a friend and were recorded at a 44.1 kHz sampling rate in a quiet room. All speakers were males to eliminate the effect of gender. Figure 1 illustrates the *F1 × F2* of CG and English (RP) vowels as produced by native speakers of these varieties. Table 1 and Table 2 present the durations and standard deviations (*SD*) of CG and English (RP) vowels, respectively.

The prediction of the classification of English vowels in terms of L1 CG categories was conducted with the use of *Linear Discriminant Analysis* (LDA) [48]. LDA was run in R [49] using the *MASS* package [50]; Strange and colleagues [38] and Gilichinskaya and Strange [51] employed the same process. LDA tests how well the L2 vowels fit with the center of gravity of the input corpus tokens, providing a predicted probability of how each L1 vowel will be classified in terms of the speakers’ L1 categories [52]. We trained one model on the *F1* and *F2* midpoint values and the average duration of CG vowels /i e a o u/. The cross-validation method revealed that the trained model yielded 97% correct classification, meaning that it would signal accurate classification accuracy. We then fed the model with the data of the test model, which included the midpoint *F1* and *F2* values as well as durations of English vowels to quantify the classification predictions. Table 3 presents the classification of English vowels /ɪ/ and /iː/ in terms of CG vowel categories.

LDA showed that English /ɪ/ was classified in terms of CG /i/ with a probability of 0.8, while the same vowel was classified with a probability of 0.2 as CG /e/. English /iː/ was classified with a probability of 1.0 in terms of CG /i/. So, both vowels were classified with a high probability in terms of CG /i/. According to UPM’s framework, English /ɪ/–/iː/ comprises a *completely overlapping contrast*, which is predicted to be discriminated in a poor manner.

## 3. The Perceptual Study

### 3.1. Methodology

#### 3.1.1. Participants

Ten male speakers of CG participated in the perceptual and production tests. Their age range was 21–37 (*M_age_* = 31.7, *SD* = 5.44) and they were born and raised in Cyprus. The participants originated from moderate-income families and reported that they had never lived in an English-speaking country for more than some months. According to their self-reports in an online questionnaire, they were advanced speakers of English (B2–C1 levels) with an average English learning onset age of 8.4 (*SD* = 0.84). They used English for 0.3 h per day on average (*SD* = 0.48) and they received English input for 1.5 h per day on average (*SD* = 0.85). Another 10 native English (RP) speakers (*n*_females_ = 5) in the age range of 24–42 (*M*_age_ = 31.4, *SD* = 6.02) served as controls. These speakers permanently resided in the UK. All participants had healthy vision and hearing and had never experienced any cognitive or language disorders.

#### 3.1.2. Stimuli

The stimuli of the perceptual tests consisted of English vowels /ɪ/ and /iː/ embedded in a monosyllabic /hVd/ word context. The words were part of the carrier phrase “They say hid/heed now”. The carrier phrases were produced twice by three adult female English native speakers at a 44.1 kHz sampling rate. The productions were normalized for peak intensity in Praat [53].

#### 3.1.3. Procedure

All participants completed the tests individually in sound-attenuated rooms. The classification test was prepared in a Praat script that was presented to the participants through a PC monitor. The participants were instructed to sit in front of the monitor and listen to the stimuli through the PC loudspeakers. Then, they were asked to click on the script label that was acoustically the most similar exemplar to the vowel they heard. The labels included the Greek orthographic representation of the 5 CG vowels, namely, “ι”, “ε”, “α”, “ο”, and “ου”. This is because Greek is orthographically transparent and vowels can be reliably presented using orthography, as seen in [54]. The speakers classified a total number of 48 trials (3 speakers × 2 vowels × 2 instances × 2 repetitions + 24 filler words). The interval between a click and the presentation of the next trial was 500 m.s. The participants were advised to provide rapid responses to the script. No feedback was given and there was not any option for repetition of the acoustic stimuli. Prior to the main experiment, participants completed a familiarization test with four test items. The classification test lasted about 10 min for each participant.

After the classification test, participants completed an AXB test in which they discriminated the target vowel contrast. The test was scripted in Praat and included the labels “A”, “X”, and “B”. The participants listened through the headphones to a triad of vowels from the PC loudspeakers and were asked to choose whether the middle vowel (X) was the same as the first (A) or the second vowel (B) by clicking on the appropriate label. Each vowel pair appeared in four possible configurations, namely, AAB, ABB, BBA, and BAA, and they discriminated a total number of 48 items (4 trials × 3 repetitions × 2 voices × 1 contrast + 24 filler words); we used tokens from two out of three speakers in this test. The X token was always acoustically different from the A and B tokens to avoid a solely auditory decision. The interstimulus interval was 1 s and the intertrial interval 500 m.s. There was a 4-trial familiarization test prior to the main test. Each participant required approximately 10 min to complete the test.

### 3.2. Results

#### 3.2.1. Classification Test

According to the results of the classification test, both English **/**ɪ/ and /iː/ were mostly classified as above chance responses in terms of CG /i/ with 0.86 and 0.98 probabilities, respectively; thus, English /ɪ/–/iː/ is a completely overlapping contrast since both contrast L2 members share the same L1 above chance response (above chance responses are those selected more often than chance. The chance score is found by dividing 100% (or 1.0) by the total number of available L1 labels on the script, here, five. So, the chance score is 20% (or 0.20) (100/5). L2 vowels classified in terms of an L1 sound with a percentage of ≤20% are considered below chance responses. However, if the classification percentage was >20%, then a one-sample *t*-test against the chance score would apply to find out whether the L1 response was above (*p* < 0.05) or below chance (*p* > 0.05)). It is expected that the contrast will yield poor discrimination. Table 4 shows the results of the classification test.

#### 3.2.2. Discrimination Test

**Figure 2:**
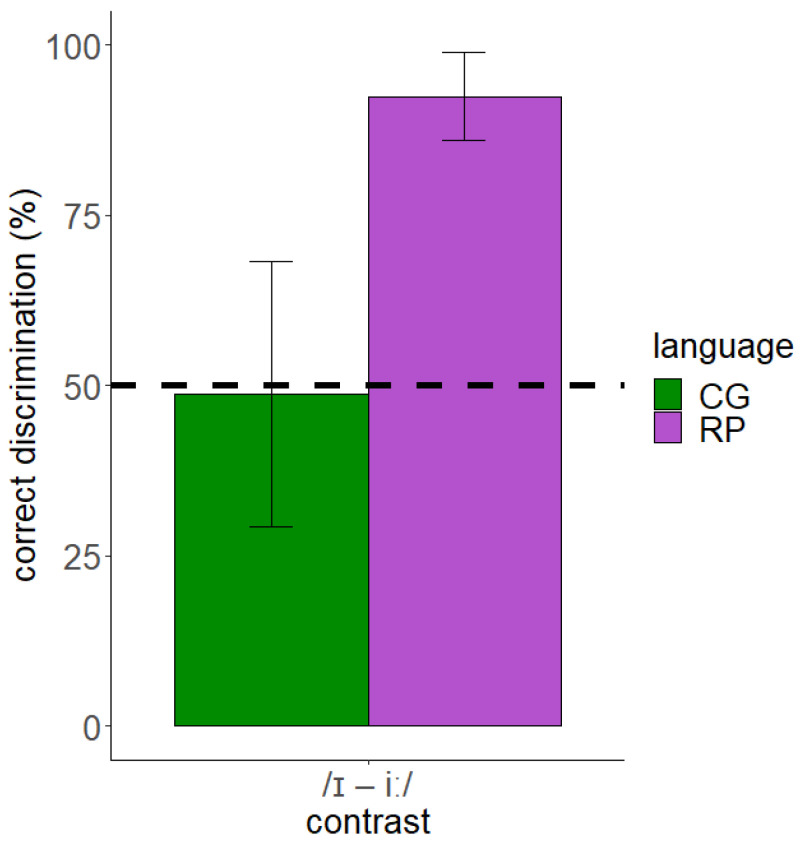
Discrimination accuracy of the English /ɪ/–/iː/ contrast by CG and RP speakers (dashed line shows chance performance).

The results of the discrimination test showed that the discrimination of English /ɪ/–/iː/ was more accurate for English native speakers than CG speakers with the latter scoring poorly (<50%). Figure 2 illustrates the discrimination accuracy of this contrast by both CG and English (RP) speakers.

The data of the discrimination test were analyzed through a *Bayesian regression model* in R using the *brms* package [55]. We used weakly informative priors, adjusting the model’s parameters as follows: student’s *t*-distribution: 3 degrees of freedom, mean: 0, and standard deviation: 2.5 [56,57]. We also used the *Bernoulli* distribution since the dependent variable response was dichotomous (0 = incorrect, 1 = correct). For hypothesis testing, we considered the Evidence Ratio (ER) and Posterior Probabilities (PP). According to Jeffreys [58], an ER of >10 provides strong evidence for a hypothesis, while an ER of <0.1 provides strong evidence against a hypothesis. We checked the hypothesis of CG < RP (the discrimination accuracy of CG speakers will be lower than the discrimination accuracy of English speakers). The results showed that there was strong evidence for this hypothesis (*β* = −2.76, *SE* = 0.62, *ER* = inf., *PP* = 1). Additionally, we examined whether the discrimination of the contrast by CG and English speakers is above chance. The findings indicated strong evidence for above chance discrimination of the contrast by English speakers (*β* = 2.70, *SE* = 0.53, *ER* = inf., *PP* = 1), but no such evidence for CG speakers (*β* = −0.06, *SE* = 0.32, *ER* = 0.69, *PP* = 0.41). Therefore, CG speakers presented with non-native-like discrimination of English /ɪ/–/iː/ and poor discrimination abilities.

## 4. The Production Study

### 4.1. Methodology

#### 4.1.1. Participants

The same participants of the experimental group who completed the perceptual test also completed the production test; the latter test took place after the former for all participants. The values of the control group were the same as those used in LDA.

#### 4.1.2. Stimuli

The stimuli were provided in a written form and included English vowels /ɪ/ and /iː/ embedded in monosyllabic /hVd/ words. The vowels were included in the carrier phrase “They say hid/heed now”, which was written using standard English orthography.

#### 4.1.3. Procedure

The test took place in sound-attenuated rooms and participants were tested individually. They were instructed to take a seat in front of a PC monitor and they were presented with the carrier phrases in PowerPoint slides. The phrases appeared in random order and participants were told to pronounce them as if speaking to a friend. Their productions were recorded through a professional audio recorder at a 44.1 kHz sampling rate and saved as wav files with a resolution of 24 bits. The participants pronounced a total number of 16 items each (2 vowels × 4 repetitions + 8 filler words).

#### 4.1.4. Acoustic Analysis

The productions were sent to Praat for speech analysis. The spectrograms and waveforms were used to decide the target vowels’ boundaries for the quantification of vowel duration and formant frequencies (*F1*, *F2*). For the purpose of this study, we only considered the values of English vowels /ɪ/ and /iː/. The following configurations were adjusted for the generation of tracks: windows length: 0.025 m.s, pre-emphasis: 50 Hz, and spectrogram view range: 5500 Hz (following [59]). The end of the noise of the first consonant /h/ and the onset of the vowel (V) in the stimuli, respectively, indicated the beginning of the vowel’s acoustic analysis. The end of the vowel (V) and the beginning of the noise of the second consonant /d/ indicated the vowel’s last point. The vowel formants were measured at their midpoint (50%). The vowel durations were extracted manually through the labeling of the initial and final points of each vowel token. The duration of the vowels was estimated from the measurement of the interval between the starting and ending point of the vocalic part. The normalization of *F1* and *F2* was produced using the *Lobanov* method in R (*vowels* package; [60]).

### 4.2. Results

**Figure 3:**
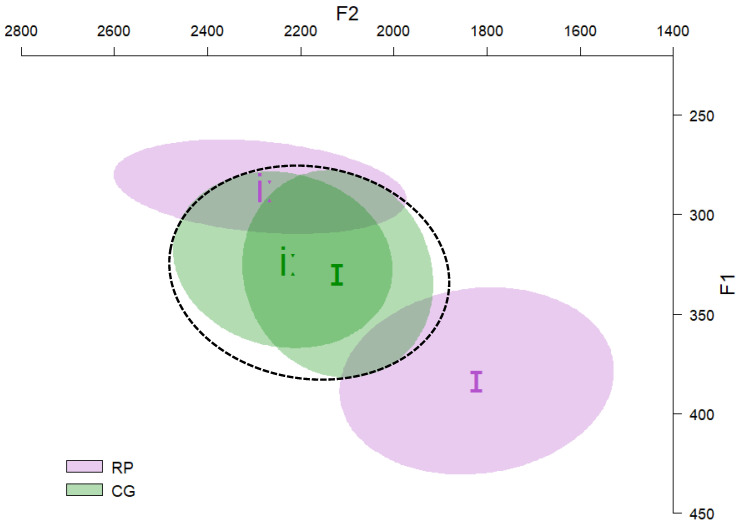
*F1 × F2* of English /ɪ/ and /iː/ as produced by CG and RP speakers.

**Figure 4:**
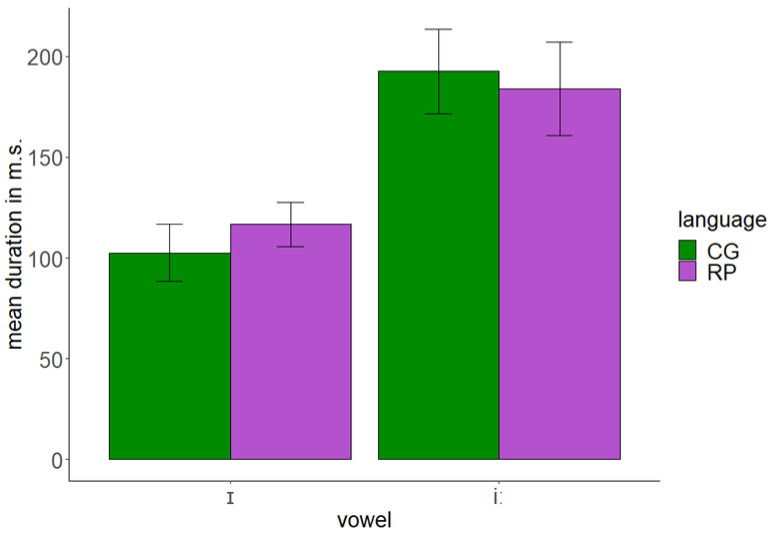
Duration of English /ɪ/ and /iː/ as produced by CG and RP speakers.

The results of the production test indicated that English /iː/ of CG speakers was acoustically close to the same vowel of English speakers. In contrast, English /ɪ/ of CG speakers was acoustically between /iː/ and /ɪ/ of English speakers. Furthermore, there was an important overlap between the two English vowels produced by CG speakers. In addition, CG speakers produced the English vowels with similar durations to native English speakers. Figure 3 and Figure 4 present the *F1 × F2* and duration of English /ɪ/ and /iː/ as produced by CG and English (RP) speakers.

We fitted *Bayesian regression models* in R to analyze the production data. We used weakly informative priors, that is, student’s *t*-distribution with 3 degrees of freedom, a mean of 0, and a standard deviation of 2.5. We also used the *Poisson* distribution since the dependent variable response is continuous. The hypothesis testing examined whether *F1/F2*/ duration CG = *F1/F2*/ duration RP for each vowel and whether *F1/F2*/duration /ɪ/ = *F1/F2*/duration /iː/ for each language. The findings demonstrated that there was strong evidence that the *F1* of /ɪ/ as produced by CG speakers differed from the *F1* of the same vowel as produced by English speakers (*β* = −0.15, *SE* = 0.03, *ER* = 0, *PP* = 0), while there was no evidence for equality between the *F1* of /iː/ produced by CG vs. English speakers (*β* = 0.12, *SE* = 0.03, *ER* = 0.50, *PP* = 0.33), meaning that CG speakers produced that vowel with a higher *F1*. There was strong evidence that the *F1* of /iː/ was equal to the *F1* of /ɪ/ as produced by CG speakers (*β* = −0.02, *SE* = 0.01, *ER* = 80.88, *PP* = 0.99), while there was strong evidence that the *F1* of these two vowels differed in English speakers (*β* = −0.29, *SE* = 0.02, *ER* = 0, *PP* = 0). In addition, there was strong evidence that the *F2* of /ɪ/ as produced by CG speakers differed from the *F2* of the same vowel as produced by English speakers with the former having a higher *F1* than the latter (*β* = 0.14, *SE* = 0.03, *ER* = 0, *PP* = 0), while there was strong evidence that the *F2* of /iː/ as produced by CG speakers was equal to the *F2* of the same vowel as produced by English speakers (*β* = −0.03, *SE* = 0.03, *ER* = 90.56, *PP* = 0.99). Additionally, there was strong evidence that the *F2* of /ɪ/ and /iː/ differed in both CG (*β* = 0.05, *SE* = 0, *ER* = 0, *PP* = 0) and English speakers (*β* = 0.23, *SE* = 0.01, *ER* = 0, *PP* = 0). With regard to duration, there was only weak evidence that there were similar durations for English /ɪ/ between CG and English speakers (*β* = −0.13, *SE* = 0.04, *ER* = 2.34, *PP* = 0.70), while there was strong evidence that both CG and English speakers produced English /iː/ with similar durations (*β* = 0.05, *SE* = 0.04, *ER* = 56.07, *PP* = 0.98). Finally, there was strong evidence that the duration of /ɪ/ and /iː/ differed in both CG (*β* = 0.63, *SE* = 0.02, *ER* = 0, *PP* = 0) and English speakers (*β* = 0.46, *SE* = 0.04, *ER* = 0, *PP* = 0).

## 5. Discussion

The present study investigated the perception and production of English /ɪ/ and /iː/ by adult CG speakers of L2 English. The predictions were initially developed through a crosslinguistic acoustic analysis of L1 and L2 sounds. The participants performed both a classification and a discrimination test. The former test served as the base for the development of the prediction regarding the discriminability of the contrast within the framework of the UPM model. The tests ran in a controlled environment using a Praat script. The participants also completed a production test in which they uttered the target vowels. The analysis of the production output was performed in Praat. The results of the discrimination and the production tests were analyzed with the use of Bayesian regression models.

The findings showed that CG speakers of L2 English perceive both English vowels /ɪ/ and /iː/ as instances of CG /i/, although these two L2 vowels differ in both spectral and duration properties. This is consistent with the results of many studies, which investigate the perception of this contrast by speakers whose L1 does not have this distinction (e.g., [33,34]). Due to the dense vowel system of their L1 (/i e a o u/) and as long as their L1 does not include this contrast, speakers seek to accommodate these L2 vowels to the acoustically closer exemplars of their L1. Although both vowels were mostly classified as CG /i/, some tokens of English /ɪ/ were perceived as belonging to CG /e/. This pattern was also observed in other studies with Spanish speakers (e.g., [61,62]). The simple explanation is that English /ɪ/ shares spectral characteristics with both CG /i/ and /e/, lying between these two CG vowels in the vowel space (see Figure 1).

The discrimination test revealed that CG speakers could not distinguish the English /ɪ/–/iː/ contrast in a native-like manner since there was strong evidence that the discrimination accuracy of controls was better. Additionally, no evidence for above chance performance was found for CG speakers, showing that they discriminated this contrast only poorly. This difficulty was initially projected by LDA on the basis of crosslinguistic acoustic similarity. Agreeing with the results of previous studies (e.g., [51,52]), LDA predicted with success both the classification and the discrimination of English /ɪ/–/iː/. The classification test, which relied on the framework of the UPM model, also provided a prediction about the discrimination of the contrast based on crosslinguistic *perceptual similarity*. English /ɪ/–/iː/ formed a completely overlapping contrast since both contrast members were classified as above chance responses in terms of the same L1 phonetic category. UPM argued that such a type of contrast would yield poor discrimination due to the high degree of perceptual overlap between the L2 sounds and the single L1 sound. Indeed, the result verified UPM’s hypothesis. We can argue that *perceptual overlap* is a good metric for the estimation of discrimination accuracy; for other studies that used similar techniques, see Faris and colleagues [63,64] and Levy [65]. One significant question that arises is why this contrast was so challenging for CG speakers since they do have adequate knowledge of English. While some studies report this difficulty in the initial stages of L2 learning (e.g., [34,62]), the findings of this study show that such difficulty is also evident in more proficient speakers of the L2. Perhaps this can be explained by the fact that the participants of the study are not very active users of English (i.e., they do not use the L2 that much in their daily life) and neither have ever been exposed for a long period of time to L2 naturalistic stimuli (e.g., by residing in an English-speaking country) nor they live in a country where the L2 is dominant.

The production of English /ɪ/ and /iː/ by CG speakers was non-native. Specifically, all acoustic parameters under investigation (*F1*, *F2*, duration) for English /ɪ/ differed from the corresponding acoustic parameters of native speakers. In addition, the *F1* of English /iː/ as produced by CG speakers differed from the corresponding production of English speakers, whereas the *F2* and duration were similar. So, even in this case, the production was non-native since it differed from the native production in one acoustic parameter. Furthermore, in the production test, CG speakers produced English /ɪ/ and /iː/ with some overlapping acoustic parameters. For example, the *F1* of the two vowels was similar, demonstrating the speakers’ difficulty in the recognition of the vowels’ spectral characteristics. Additionally, the fact that CG speakers employed different durations to produce /ɪ/ and /iː/ shows that they are *aware* of the temporal difference between the two English vowels. They may have used this information to pronounce them as accurately as possible. Several studies have shown that Spanish speakers use durational cues to distinguish English /ɪ/–/i/ [66,67]. Specifically, this pattern was observed for stages 0–2 of L2 learning (see [68]). As Spanish and CG have a similar vowel system, perhaps, the learning of this contrast by the participants of this study falls within these developmental stages. Finally, in the lens of UPM’s framework, L2 English /ɪ/ and /iː/ can be characterized as *disoriented* since they are not acoustically close to the productions of native speakers. Several factors determine the orientation such as L1–L2 use, exposure to the L2, phonetic training, etc.

The results of this study allow for drawing some general conclusions about the perception–production relationship. This relationship is reflected in the following way. As /ɪ/ and /iː/ comprise two distinct categories in English, one would expect that their main acoustic parameters would maintain a consistent distance from each other in order to be produced distinctly. While this is the case for native /ɪ/ and /iː/, L2 /ɪ/ and /iː/ acoustically overlap in terms of *F1* and, in general, their acoustic spectral distance is smaller compared to the corresponding distance of native productions. This practically means that the members of this completely overlapping contrast, which is the most difficult according to UPM, will hardly be produced with native or near-native properties; they may be produced with similar acoustic properties. Therefore, a (near-) unified *interlinguistic* vowel exemplar is activated, which represents both L2 English vowels (see Figure 3). Although UPM is mainly a perceptual model, its predictions can be extended to speech production as well. However, more studies are needed to generalize this conclusion.

## 6. Conclusions

English /ɪ/–/iː/ is a challenging contrast for CG speakers both in the perception and the production domains—it can neither be distinguished adequately nor produced in a native-like manner even by experienced speakers of L2 English. It might be that this contrast carries a universal marker of difficulty for speakers with L1s that have fairly simple vowel systems, which do not possess this contrast. In addition, the hypotheses developed by the UPM model can be useful for the predictions of the discriminability of L2 sound contrasts and can be extended to speech production. Future studies may explore the acquisition of a larger number of English contrasts by CG speakers. They may also examine other acoustic properties such as *F0* and *F3* and consider the role of linguistic and extralinguistic factors in L2 speech acquisition.

## Figures and Tables

**Table 1:** Duration of CG vowels.

Vowel	Duration (m.s.)	*SD*
/i/	111	10.2
/e/	129	11.1
/a/	146	15.6
/o/	143	12
/u/	124	15.8

**Table 2:** Duration of RP vowels.

Vowel	Duration (m.s.)	*SD*
/ɪ/	117	11.1
/iː/	184	23.2
/e/	135	11.5
/ɜː/	201	10.1
/æ/	163	15.8
/ɑː/	215	6.5
/ʌ/	127	10.7
/ɒ/	129	9.7
/ɔː/	206	13.3
/ʊ/	191	26.1
/uː/	117	14.8

**Table 3:** Classification results of the LDA analysis for English vowels /ɪ/ and /iː/. Dark gray cells represent the L1 responses with the highest probability.

Vowels		CG				
		i	e	a	o	u
RP	ɪ	0.80	0.20			
	iː	1.00				

**Table 4:** Results of the classification test. Dark gray cells represent the L1 responses with the highest probability. Bold values indicate above chance performance.

Vowels		CG				
		i	e	a	o	u
RP	ɪ	**0.86**	0.14			
	iː	**0.98**	0.02			

## Data Availability

Not applicable.
